# Clinical Outcomes of Refeeding Syndrome: A Systematic Review of High vs. Low-Calorie Diets for the Treatment of Anorexia Nervosa and Related Eating Disorders in Children and Adolescents

**DOI:** 10.7759/cureus.39313

**Published:** 2023-05-21

**Authors:** Emmanuel M Mosuka, Anushree Murugan, Abhinav Thakral, Mbelle C Ngomo, Sushil Budhiraja, Rosemarie St. Victor

**Affiliations:** 1 Pediatrics, Brookdale University Hospital Medical Center, New York, USA; 2 Medicine, Université de Yaoundé, Faculté de Médecine et des Sciences Biomédicales, Yaoundé, CMR

**Keywords:** re-alimentation, hypophosphatemia, low calorie diet, high calorie diet, weight gain, refeeding syndrome, refeeding, anorexia nervosa

## Abstract

Over the years, the standard of care for re-alimentation of patients admitted for the treatment of anorexia nervosa (AN) has been a conservative or cautious approach described as "start low and go slow." These traditional refeeding protocols advocate for a low-calorie diet that restricts carbohydrates, with the primary goal of hypothetically lowering the risk of refeeding syndrome (RFS) and its complication. However, no consensus exists for the optimal inpatient approach to refeeding children and adolescents with AN. There is still some disagreement about what constitutes an ideal pace for nutritional rehabilitation. Varying treatment protocols have emerged across the globe, often reflecting the preferences and biases of individual practitioners and contributing to the lack of a universally accepted protocol for refeeding in AN. Although it is widely accepted that low-caloric refeeding (LCR) is safe for inpatient treatment of AN, this strategy has been shown to have several significant drawbacks, leading to increased criticism of the LCR method. Research from the last decade has led to calls for a more aggressive refeeding protocol, one that suggests a higher caloric intake from the offset. As a result, this research aimed to conduct a systematic review of the existing literature on strategies for refeeding hospitalized pediatric/adolescent patients with AN and related eating disorders. We aimed to compare high-caloric refeeding (HCR) and LCR in terms of weight gain, length of stay, and risk of RFS. We conducted a thorough search of medical databases for abstracts published in English, including Google Scholar, PubMed, and MEDLINE, to find relevant studies published between 2010 and February 2023. Our focus was on articles that evaluated high versus low refeeding protocols in children and adolescents hospitalized for treating AN and related eating disorders. Only articles that reported on at least one of the outcome variables of interest, such as hypophosphatemia, weight gain, RFS, or length of hospital stay, were considered. This review included 20 full-text articles published in the last decade on the HCR protocol in children and adolescents, with a total sample size of 2191 participants. In only one of the 20 studies did researchers find evidence of a true clinical case of RFS. We, therefore, found no evidence that HCR increased the risk of RFS in adolescents, even in those with a very low body mass index (BMI). However, evidence suggests a lower BMI at the time of hospital admission is a better predictor of hypophosphatemia than total caloric intake. In conclusion, based on the evidence from this review, a high-caloric diet or rapid refeeding in children/adolescents suffering from AN may be both safe and effective, with serial laboratory investigations and phosphate supplementation as needed. Hence, more research, particularly, randomized controlled trials, is required to help shape an evidence-based refeeding guideline outlining target calorie intakes and rates of advancement to assist clinicians in the treatment of adolescents with AN and related eating disorders.

## Introduction and background

Anorexia nervosa (AN) is a type of eating disorder that, even with treatment, has the potential to be fatal [1]. It has the highest death rate of all mental disorders [2,3] and is characterized by severe dietary restrictions and extreme anxiety about gaining weight [4,5]. The most common age range for the onset of AN is between the ages of 15 and 19 years [6,7], with the majority of affected patients being female adolescents [8-10]. It is most severe in adolescents who, due to caloric restriction, excessive exercise, and other behaviors, can quickly become malnourished during a crucial period of growth and development [11]. This emphasizes the significance of hospital admission for medically unstable children and adolescents suffering from severe malnutrition due to AN. The mainstay of treatment includes nutritional support, psychological therapy, and inpatient care [12]. To stabilize the patient's health and undo the effects of malnutrition, nutritional rehabilitation is crucial in the treatment of AN. It has also been reported that a patient's weight gain during hospitalization for AN is an important factor in predicting weight recovery a year later, as well as in facilitating initial stabilization and reducing the risk of rehospitalization [13,14].

The first and most important step toward recovery from AN is reintroducing feeding or re-nutrition [15,16]. However, no consensus guidelines exist for the optimal inpatient approach to refeeding children and adolescents with AN. There is still some disagreement about what constitutes an ideal pace for nutritional rehabilitation. Due to the lack of a universally accepted protocol for refeeding in AN, varying treatment protocols have emerged across the globe, often reflecting the preferences and biases of individual practitioners. The consensus is that the need for adequate nutrition to regain weight and achieve medical stability must be weighed against the risks of refeeding syndrome (RFS) and its potentially fatal complications such as cardiac arrest, arrhythmia, coma, seizures, and sudden death [17,18]. Over the years, the standard of care for re-alimentation in AN has been a more conservative approach popularly described as "start low and go slow." These traditional refeeding guidelines advocate for a low-calorie diet (LCD) that restricted carbohydrates, with the primary goal of hypothetically lowering the risk of RFS and its complication [17]. For instance, the standard of care for in-patient refeeding in the United States has been an LCD beginning at approximately 1200 kcal/day and increasing by 200 additional calories every alternate day [19,20]. In other parts of the world, such as Australia, the United Kingdom, and Europe, daily calorie intakes as low as 200-600 kcal have been advised [20-22].

Varying treatment protocols have emerged across the globe, often reflecting the preferences and biases of individual practitioners and contributing to the lack of a universally accepted protocol for refeeding in AN. Although it is widely accepted that low-caloric refeeding (LCR) is safe for inpatient treatment of AN, this strategy has been shown to have several significant drawbacks, leading to increased criticism of the LCR method. Recently, a growing body of evidence suggests that it may be associated with poor initial weight gain, early weight loss (first one to two weeks), and extended hospital stays, which add to the financial and emotional burdens on families and the healthcare system [23,24]. In addition, some argue that LCR has not eliminated the incidence of RFS. Moreover, studies have shown that the severity of pretreatment malnutrition is a more significant risk factor for refeeding hypophosphatemia (RH) than either calorie intake or rate of weight gain [25-27]. Moreover, newer research from the last decade has led to calls for a more aggressive refeeding protocol, one that suggests a higher caloric intake from the offset, typically above 1400 kcal/day, and a rapid increase in that amount [28-33].

Unfortunately, there is a shortage of research addressing whether or not refeeding at higher calorie levels is risky for severely undernourished adolescents who have AN and associated feeding disorders. Garber et al. made two critical observations in their systematic review of the different approaches to refeeding in hospitalized patients with AN in 2016 [16]. They concluded that higher calorie refeeding is both feasible and safe with close medical supervision and with the correction of electrolyte abnormalities. Furthermore, a systematic review that was conducted in 2012 by O'Connor et al. demonstrated that the severity of malnutrition at the beginning of the re-nutrition process was a more significant factor in determining RH than total energy intake [34]. On the other hand, a randomized controlled trial conducted by Golden et al. in 2021, comparing LCD and high-calorie diet (HCD) with initial calorie intakes of 1400 kcal/day and 2000 kcal/day, respectively, found no significant differences in weight gain, length of hospital stay, or rate of occurrence of RFS [35].

Consequently, the goal of this research was to carry out a systematic review of the existing literature on strategies for refeeding hospitalized pediatric/adolescent patients with AN and related eating disorders. We aimed to compare high-caloric refeeding (HCR) and LCR in terms of weight gain, length of stay, and risk of RFS.

## Review

Method

Protocol

This systematic review followed the latest Preferred Reporting Items for Systematic Reviews and Meta-Analyses (PRISMA) guidelines [36].

Search Strategy

We conducted a thorough search of medical databases for abstracts published in English, including Google Scholar, PubMed, and MEDLINE, to find relevant studies published between 2010 and February 2023. The following keywords were included in the search strategy: refeeding syndrome, anorexia nervosa, eating disorder, re-nutrition, high caloric diet, low caloric diet, and hypophosphatemia. After locating and retrieving all relevant studies, the reference lists of each of those studies were reviewed to identify any additional relevant studies. The references were imported into Mendeley Web Importer (Elsevier, Amsterdam, Netherlands), where they were compared by author, year, title, and reference type. This enabled the removal of any discovered duplicates.

Eligibility Criteria

We focused on articles that evaluated high versus low refeeding protocols in children and adolescents hospitalized for the treatment of AN and related eating disorders. Articles were included if they were published within the last decade. Furthermore, articles were only included if they outlined a clear refeeding protocol that was reproducible, both in terms of caloric intake and rate of administration. Lastly, articles were included if they reported on at least one of the outcome variables of interest, such as hypophosphatemia, weight gain, RFS, or length of hospital stay (LOS).

We excluded studies solely conducted in non-pediatric/adolescent populations, case studies, and review articles. Furthermore, articles with participants who had comorbidities, such as cancer, or other severe medical disorders, such as renal, cardiac, or hepatic failure, were excluded because these could affect our outcome variables.

Study Selection

Two reviewers (EM and AM) conducted an initial screening, with any discrepancies resolved by our supervisor (RV). Following an initial screening, full-text publications that met our inclusion criteria were added to the database.

Study Population 

**Figure 1:**
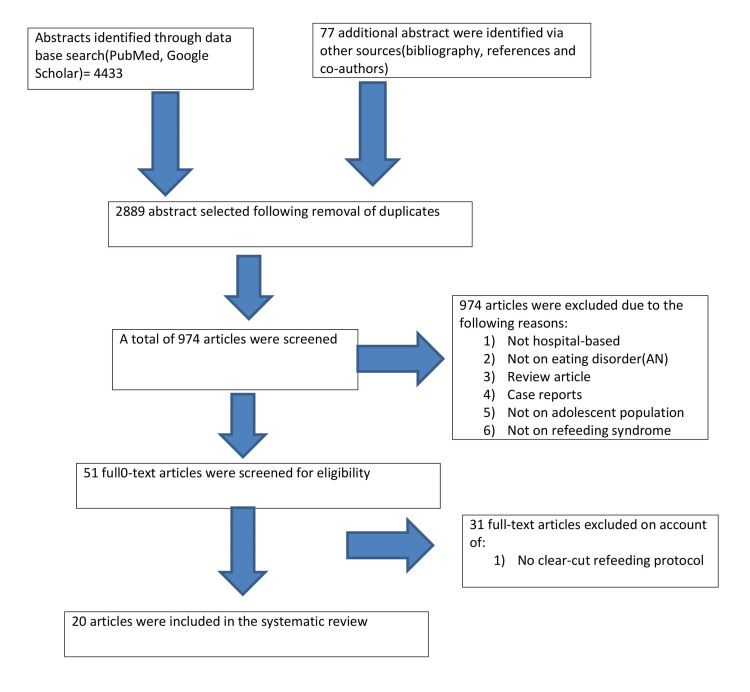
PRISMA flowchart describing the data collection and study selection processes PRISMA: Preferred Reporting Items for Systematic Reviews and Meta-Analyses; AN: anorexia nervosa.

Figure 1 is a flowchart that depicts the process of selecting appropriate articles for this study. Initial searches of electronic databases yielded 4433 references. A review of the bibliography and reference lists of related studies yielded 77 additional articles. After duplicates were removed, 2889 abstracts were included in the screening process. Initial screening based on inclusion and exclusion criteria resulted in the exclusion of 1915 abstracts. We excluded studies conducted solely in non-pediatric/adolescent populations, articles published more than 12 years ago, case studies, and review articles. We also excluded studies involving malnourished patients who did not have an underlying eating disorder. Following this preliminary selection, we were left with 51 abstracts, the full texts of which were retrieved and re-examined against the inclusion and exclusion criteria. Thirty-one additional studies were excluded because the refeeding method and outcomes were not clearly defined.

Study Characteristics 

Table 1 depicts the varied characteristics of the 20 included studies, more than half of which were done in the United States and Australia. Almost 95% of the papers that satisfied our inclusion criteria were published during the last decade, and the majority (80%) were either randomized controlled trials (RCTs) or retrospective studies (cohort and chart reviews). Ten studies compared low-calorie diets to high-calorie diets [26,35,37-45], whereas the remaining studies only looked at high-calorie diets. There was no standard protocol used. We observed a wide range of refeeding strategies, with varying initiation caloric rates and daily rates of caloric advancement. The average initial refeeding intake ranged from 740 to 1400 for LCR and 1400 to 3000 for HCR. Furthermore, most studies included serial laboratory investigations and phosphate monitoring. In five studies [40,46-49], phosphate supplementation was given routinely to all patients as a measure of reducing the risk of RS; however, in other studies, phosphate supplementation was given as needed depending on laboratory readings during admission [26,44,50,51]. In terms of bias assessment, there was a high risk of bias due to the observational/retrospective study designs utilized in the majority of studies, as well as a high risk of attrition bias due to incomplete outcome reporting.

**Table 1:** General characteristics including total caloric intake, rates of hypophosphatemia, refeeding syndrome, weight gain, and length of hospital stay AN: anorexia nervosa; HCR: high-caloric refeeding; LCR: low-caloric refeeding; mBMI: mean body mass index; RCT: randomized controlled trial; EBW: expected body weight; NG: nasogastric; LOS: length of hospital stay.

Author and year of publication	Study design and country where the study was done	Admission BMI or degree of malnutrition	Characteristics of participants	Length of follow-up	Refeeding protocol/method	Rate of weight gain and length of stay	Incidence of refeeding syndrome/hypophosphatemia
Dalenbrook et al. (2022) [52]	Retrospective chart review on HCR refeeding protocol. Germany	Median BMI was 13.1 ± 1.1 (range = 10.2–15.0), % mBMI was 62.1 ± 6.0%	120 adolescents aged 12 to 20 years with body mass index (BMI) < 15 kg/m^2^.119 (99.2%) were female	4 weeks	HCR: starting at 2000 kcal/day and increasing by 200 kcal daily	0.76 ± 0.22 kg/week. Total weight gain of 3.00 ± 1.92 kg in 4 weeks	No cases of refeeding syndrome, and there was an increased likelihood to receive phosphate supplementation. 9 patients (7.5%) developed mild hypophosphatemia, and none developed RFS. Correlation between degree of malnutrition (initial BMI) with hypophosphatemia
Schlapfer et al. (2022) [45]	Retrospective chart review comparing HCR vs. LCR refeeding method. USA	N/A	291 adolescents aged 12 to 21 years. LCR (n = 137) versus HCR (n = 154)	Until medical stability	LCR began at 1300 kcal/day while HCR at 1400 kcal/day	The HCR group's length of stay was significantly shorter than the LCR group (p = 0.006). No significant difference in weight gain	Six (4.4%) patients in the LCR group and four patients (2.6%) in the HCR group had severe refeeding syndrome
Draffin et al. (2022) [41]	Randomized controlled trial comparing HCR vs. LCR refeeding method. Australia	Median BMI of 92.4% (LCR) and 86.5% (HCR)	LCR (n = 12) versus HCR (n = 11)	7 days admission follow-up	Most participants were on 2000—2500 kcal/d; LCR had 40% of total energy from carbohydrates while the HCR group had 50–60% of total energy from carbohydrate	Weight gain in the first week was significantly higher in the HCR (1.4 kg/wk ± 0.5) compared to the LCD (0.6 kg/wk ± 0.9), with a p-value of 0.03	No incidence of refeeding syndrome in either group
Golden et al. (2021) [35]	Multicenter RCT comparing HCR vs. LCR refeeding method. USA	Median BMI ≥60%	111 adolescents (HCR/60 vs. LCR). Participants had a mean age of 16.4 years. The mean percentage of mBMI was 84.9%	12 months (10 days of admission)	HCR (2000 kcals per day, increasing by 200 kcals per day) or LCR (1400 kcals per day, increasing by 200 kcals every other day)	Both groups gained weight over time with no significant difference. The average length of stay was shorter by 4 days for the HCR group	At one year, there was no difference in clinical remission or medical rehospitalization between HCR and LCR
Garber et al. (2021) [38]	Multicenter RCT comparing HCR vs. LCR refeeding method. USA	Median BMI ≥60%	111 adolescents (HCR/60 vs. LCR). Participants had a mean age of 16.4 years. The mean percentage of mBMI was 84.9%	12 months (10 days of admission)	HCR (2000 kcals per day, increasing by 200 kcals per day) or LCR (1400 kcals per day, increasing by 200 kcals every other day)	N/A	The HCR group was medically stable 3 days faster. 5% developed hypophosphatemia
Davis et al. (2021) [44]	A case-control study comparing HCR vs. LCR refeeding method. Singapore	% mBMI was 73.2	125 adolescents with 61 (49%) patients in the HCR group and 64 (51%) in the LCR group	Until stable for discharge (mean length of stay was 11.9 days)	LCR (1400–1500 kcal/d, increasing every day to 200–300 kcal every 3 days. HCR (1600-1800, increasing on an average of 200–300 kcal per day to attain a final meal plan of 3000–3600 kcal per day	Participants in the HCR group had a significantly increased rate of change of % mBMI (M = 0.39, SD = 0.31) than patients in the LCR group (M = 0.12, SD = 0.43) (p < 0.001). No significant difference in LOS	No incidence of refeeding syndrome. There was an increased incidence of mild hypophosphatemia in the HCR group (HCR: 46%, LCR: 22%, p = 0.007) but no difference in rates of moderate hypophosphatemia and no cases of severe hypophosphatemia
Parker et al. (2021) [43]	Randomized control trial comparing HCR vs. LCR refeeding method. Australia	Median BMI 77–79%	A total of 24 adolescents aged 14 to 24 years	7 days	Isocaloric initial calorie intake: 14 patients on high fat/low carbohydrate versus 10 patients on standard diet (55% carbohydrate and 25% fats)	No significant difference in LOS and weight gain between groups	Reduced incidence of hypophosphatemia in the treatment group (5/14 vs. 9/10; p = 0.013) during week 1
Maginot et al. (2017) [42]	Retrospective chart review comparing HCR vs. LCR refeeding method. USA	Expected body weight: LCR = 78.7% and HCR = 81.2%	87 adolescents with a mean age of 14.4 years (8–20 years). 75.8% (66) were in the HCR group	Medical stability (average 72 hours)	HCR group's mean initial intake was 1781 kcal/day (1500-3000) versus 1185 kcal/day (1000-1300) for LCR	N/A	The risk of hypophosphatemia or hypokalemia in the first 72 hours with HCR. Higher rates of readmission are seen for LCR (40% vs. 6%). Lower admission % EBW was a stronger predictor of hypophosphatemia than initial calorie intake
Peebles et al. (2017) [50]	Retrospective chart review. USA	Median body weight of 86%	Total of 215 patients with ages from 5.8 to 23.2 years (mean of 15.3 years) and most were females (88%)	Medical stability and 4 weeks after discharge	The Children’s Hospital of Philadelphia inpatient nutritional rehabilitation protocol (HCR). The initial mean intake was 1466 kcal/d and steadily increased to a mean of 3800 kcal/d at discharge	On average, patients gained 2.5 kg during their stay, resulting in a 91% mBMI at the time of discharge compared to 81% on admission. Mean LOS 11 days	There was no incidence of full-blown refeeding syndrome. Just about 15% required phosphate supplementation
O'Connor et al. (2016) [37]	Randomized control trial comparing HCR vs. LCR refeeding method. UK	Median BMI <78%	36 adolescents aged 10 to 16 years with the majority being female (94%)	A total of 10 days of re-nutrition	HCR (18/36) group with an initial intake of 1200 kcal/day versus 800 kcal/day for LCR (18/36)	N/A	While hypophosphatemia was linked to initial BMI before treatment, initial caloric intakes had no effect
Parker et al. (2016) [46]	Retrospective cohort using HCR refeeding method. Australia	Mean BMI of 80.1%	162 adolescents aged 14 to 17 years with the majority being female (91%)	Medical stability after nutritional rehabilitation	HCR: starting at ≥2400 kcal/day for >48 hours with a mean initial caloric intake of 2611.7 kcal/day	There was an average weight gain of 2.1 kg/week with increased BMI (median BMI was 93.1% at discharge from 80.1% at admission)	There were no cases of full-blown refeeding syndrome but they did experience the following: peripheral edema (4%), hypophosphatemia (1%), hypomagnesemia (7%), and hypokalemia (2%)
Pettersson et al. (2016) [49]	Observational study. Sweden	BMI of 15.4	A total of 21 adolescents aged 16–24 years with AN	12-week follow up	HCR: the mean total daily initial caloric intake was 3264 kcal and decreased gradually during treatment to 2622 kcal	Patients on average gained 9.8 kg in 12 weeks, equivalent to 0.82 kg/week	No case of refeeding syndrome
Smith et al. (2016) [51]	Retrospective chart review. USA	Median BMI of 79.4%	129 adolescents aged 10–22 years with AN admitted for medical stabilization	Medical stability and 4 weeks after discharge	Starting at 1500 kcal, increasing by 250 kcal every day or every other day until 2500-3000 kcal by day 14	Participants had a mean weekly weight gain of 1.39 kg. Reduced LOS	No case of refeeding syndrome was noted. Although no patient had hypophosphatemia at discharge, phosphorus supplements were prescribed to 100 patients (77.5%)
Madden et al. (2015) [47]	Cohort study. Australia	EBW of 78.37%	78 adolescents (12–18 years) with AN were hospitalized for medical instability in two specialized pediatric eating disorder units	2.5 weeks for medical stabilization	The patient had a daily caloric intake of 2400–3000 kcal/day along with routine oral phosphate supplementation	All participants had a mean weight gain of 2.79 kg and 5.12 kg at 2.5 weeks, respectively	No patient exhibited hypophosphatemia, hypoglycemia, or symptoms of refeeding syndrome
Garber et al. (2013) [26]	A prospective observational study using a quasi-experimental design to compare LCR vs. HCR. USA	Median BMI of 80.1%	56 adolescents aged 9–20 years with AN	14.9 ± 9 days admission	LCR initial intake: 800–1200 kcal/day (mean of 1093 kcal) HCR: 1400–2400 kcal/day (mean of 1764 kcal)	The rate of weight gain was nearly double in the higher calorie group: 0.27 (±0.03) versus 0.14 (±0.02) kg/day (p < 0.001). The LCR group initially lost weight with no significant weight gain until day 8. The average LOS was 5.7 days shorter (p < 0.001) in the HCR group	There were no clinical cases of refeeding syndrome found, but 45% of participants (25/56) had low serum phosphorus levels. The HCR group had a greater tendency to receive phosphate supplementation (12 vs. 8, p = 0.0273)
Agostino et al. (2013) [40]	Non-RCT. USA	Ideal body weight of 82–85%	165 adolescents aged 10 to 18 years with restrictive eating disorders. Higher calorie NG refeeding protocol (N = 31) versus standard bolus meal strategy (N = 134)	Medical stability after nutritional rehabilitation (2 weeks)	LCR: initiated at 1000 to 1200 kcal/d and increased by 150 kcal/day. HCR: 1500 or 1800 kcal/day via continuous NG feeds, advanced by 200 kcal/day	The mean rate of weight gain in HCR was significantly higher (1.22 kg/week, p = 0.01) than in the LCR group (1.06 kg/week, p = 0.04). Length of stay was significantly reduced in HCR (33.8 vs. 50.9 days; p = 0.0002)	With 90% of the HCR group receiving prophylactic phosphate supplementation from admission, there was no significant difference in the rate of complications or electrolyte abnormalities
Golden et al. (2013) [39]	Retrospective cohort comparing HCR vs. LCR refeeding method. USA	Median BMI of 78.5%	310 adolescents aged 10–21 years (mean age of 16.1 ± 2.3 years) with AN, predominantly female (88.4%)	Medical stability after nutritional rehabilitation	LCR (n = 88): initiated at 720 to 1320 kcal/day. HCR (n = 222): initiated 1400 or 1800 kcal/day	LOS was significantly shorter in the HCR group 13.0 ± 7.3 days versus 16.6 ± 9.0 days (in LCR); p ≤ 0.0001. No significant difference in weight gain	There were no cases of clinical refeeding syndrome in either group
Leitner et al. (2016) [48]	Retrospective chart review on HCR plus phosphate supplementation. Canada	Median BMI of 83.5%	70 adolescents aged 10 to 18 years (mean age of 15.3 years) with a restrictive eating disorder	1 week of nutritional rehabilitation	An initial daily caloric intake of 1800 kcal/day (via NG tube) to a mean maximum caloric intake (2422 ± 170 kcal/day). Supplementation with phosphate for 7 days, which was then tapered off	N/A	No cases of refeeding hypophosphatemia. 14.7% of those on routine phosphate supplementation had mild asymptomatic hyperphosphatemia
Leclerc et al. (2013) [27]	Retrospective chart review. Canada	Ideal body weight >70%	29 adolescents aged 12–18 years (mean age of 14.7) with AN	Medical stability after nutritional rehabilitation	Starting at 1500 kcal, increasing by 250 kcal every day or every other day until 2500 kcal by day 7	Mean weight gain was 0.24 kg/day (p < 0.0001) and 1.7 kg/week	Low serum phosphate levels were reported in 1/29 participants necessitating oral supplementation on day one of the protocol. There were no cases of clinical refeeding syndrome. No case of refeeding syndrome reported
Whitelaw et al. (2010) [30]	Retrospective chart review. Australia	Mean ideal body weight of 72.9%	29 adolescents aged 12–18 years (mean age of 15.7 years) with AN	2 weeks (medical stability after nutritional rehabilitation)	Starting at 1900 kcal to 2200 kcal/day increasing to a maximum of 2700 kcal	N/A	There were no clinical cases of refeeding syndrome found, but 37% of participants had mild hypophosphatemia. % ideal body weight on admission was significantly associated with hypophosphatemia. A higher incidence of hypophosphatemia was seen in patients with an ideal body weight of less than 68%

Discussion

The conservative approach to refeeding severely malnourished patients, including those with AN, has traditionally been used in hospitals. This cautious approach of starting low and gradually increasing caloric intake has been thought to be critical in preventing or reducing the risk of RFS. The central idea behind this conservative refeeding protocol, which is not based on any empirical evidence, stems from the pathophysiology for refeeding malnourished patients, specifically the idea that reducing total caloric intake prevents insulin surge, which in turn suppresses the rapid intracellular movement of electrolytes, water, and glucose, thus preventing RFS. While there is consensus that a low-caloric approach is a safe option for the inpatient treatment of AN, this approach has been seen to have several significant drawbacks. Recently there has been mounting evidence that it may be associated with poor weight gain and extended hospital stays. Therefore, in recent years, there has been growing support for a more robust refeeding protocol.

In this systematic review, we compared the results of LCR to those of HCR, specifically looking at the rates of weight gain, hospital stays, and occurrences of RFS or hypophosphatemia.

Initial Caloric Intake and Occurrence of Refeeding Syndrome or Hypophosphatemia

Concerning the potential for hypophosphatemia and RFS, there is currently no consensus regarding the safety of a high-caloric diet re-alimentation protocol for adolescents with AN. Based on the findings of this systematic review, we believe that the concerns about HCR causing RFS have been exaggerated over time. We found no evidence that HCR increased the risk of RFS in adolescents, even in those with a very low BMI. Our review comprised a total of 20 articles published in the last decade on the HCR protocol in children and adolescents, with a total sample size of 2191 participants. In only one of the 20 studies did researchers find evidence of a true clinical case of RFS [45]. Schlapfer et al. reported a total of 10 cases of overt RFS in their 2022 study comparing HCR and LCR. Although not statistically significant, it was interesting to see that more of these cases happened in the LCR group (4.4%; 6/137) than in the HCR group (2.6%; 4/154) [45].

Furthermore, findings from our research, as illustrated in Table 1, showed that the occurrence of hypophosphatemia was highly variable with total caloric intake, making its prediction challenging. The highest incidence of hypophosphatemia was 45%, as reported by Garber et al., at an initial caloric intake of 800-1200 kcal for LCR and 1400-2400 kcal for HCR [26]. In their study, there was a slightly increased tendency to receive phosphate supplementation in the HCR group than in the LCR. Though the incidence of mild hypophosphatemia was more prevalent in the HCR group, it also occurred in the LCR group. The seemingly inconsistent presence of RH in adolescents with AN who were initiated with both high and low refeeding rates adds to the complexity of this physiological phenomenon and suggests that RH may not be completely related to energy intake. Given the inconsistency in the presentation of RH occurring at varying energy intakes (HCR and LCR), our study suggests that several factors may play a part in the incidence of RH in an adolescent with AN.

Furthermore, in some studies, patients in the HCR group were given routine phosphate supplements at the start of refeeding, resulting in no or few cases of mild hypophosphatemia [40,47-49,53]. In other studies, participants who were deemed to be at risk of developing severe hypophosphatemia based on serial labs performed during admission were given prophylactic phosphate supplementation [26,44,50,51]. This supports the notion that a rapid high-caloric feeding protocol can be safely implemented in hospitalized adolescents with AN, with close monitoring and electrolyte supplementation as needed.

Initial Mean BMI and Occurrence of Hypophosphatemia

Five studies found an association between BMI at the time of admission and the incidence of hypophosphatemia; specifically, they reported that a lower BMI at admission was a better predictor of hypophosphatemia than the initial caloric intake [30,35,37,42,44]. This was similar to the conclusion of O’Connor and Nicholls in their systematic review in 2013 on RH in adolescents with AN [34]. They included 17 publications in their article including 10 case reports, five chart reviews, and an observation study. Like our study, they found that the severity of hypophosphatemia was not influenced by total caloric intake but was directly associated with decreasing BMI on admission. Whitelaw et al.'s 2010 study was one of the very first to examine the HCR refeeding protocol. They observed an increased incidence of hypophosphatemia in HCR participants with an ideal body weight of less than 68% at the time of admission [30].

Rate of Weight Gain and Total Caloric Intake

It has been reported that the amount of weight a patient with AN gains while hospitalized is a key determinant both for early stabilization and in predicting weight recovery a year later. The findings of this study, along with other studies published in the last decade, cast doubt on the conservative "start low, go slow" feeding model. In this review, 15 studies looked at weight gain as an outcome; seven compared weight gained in the high-caloric group to that of LCR protocols [26,35,40,41,43-45], and eight looked only at the HCR protocol [27,39,47,49-53]. In 11 of these 15 studies, the HCR group's average weekly weight gain was significantly higher than that of the LCR group, ranging from 0.82 kg to 2.7 kg [26,27,40,41,44,46,47,49-52]. The most significant weight gain was reported in Garber et al.'s prospective observational study, which used a quasi-experimental design to compare LCR versus HCR refeeding methods. They observed that after one week of admission, the rate of weight gain in the HCR group was nearly double than that of the LCR group [26]. These studies demonstrated the feasibility of faster weight gain in HCR participants without an increased risk of RFS. This is an important finding that could help doctors ensure that AN patients get the nutrition they need to recover from the disease thereby preventing problems like inadequate refeeding, suboptimal weight gain, or even weight loss in the early phase of treatment. Furthermore, the sooner these patients gain weight, the better their cognitive function, which in turn leads to better engagement in additional therapy such as psychotherapy, which is required for eventual recovery from AN.

Total Caloric Intake and Length of Hospital Stay

Nine studies examined the LOS as an outcome variable, with seven finding a correlation between HCR refeeding protocol and shorter hospital stay [26,35,39,40,45,50,51], while the other two found no significant difference in length of stay between the two groups [44]. The most notable was reported by Agostino et al., who reported a statistically and clinically significant reduction in hospital stays of 17 days [40]. Given the scarcity of inpatient AN treatment facilities, which results in a lack of available beds and prohibitive costs, reducing the LOS not only saves money but also allows for the treatment of more patients. This review's findings are consistent with those of Staab et al., who conducted a prospective cohort study on 325 adult patients with a BMI less than 18 who were hospitalized for the treatment of AN in Canada [53]. They found an average LOS reduction of 21 days when using the quick refeeding approach. According to their study, each patient could expect to save $7,748 for every 13 days of LOS reduction [53].

Strengths and limitations

The greatest strength of our review is the inclusion of 20 articles, including five RCTs, as opposed to the previous two systematic reviews on this topic, which had limited RCTs and included case reports.

The main limitation of this review is that the definition of hypophosphatemia was not consistent across studies. It is possible that RH is either under or over-reported. In addition, there was no consistent definition of HCR or LCR across the articles. The final estimates of outcome variables may be impacted by the fact that what was considered HCR in some studies was LCR in others.

## Conclusions

The result of this study provides strong evidence challenging the traditional refeeding approach to managing adolescent patients hospitalized for AN. Our findings suggest that the severity of hypophosphatemia or RFS may not correlate with total caloric intake but may be influenced by the severity of malnutrition before admission. With serial laboratory investigations and phosphate supplementation, when necessary, a high-caloric diet or rapid refeeding in children/adolescents suffering from AN may be both safe and effective. In addition, HCR may shorten the LOS, which minimizes cost and improves weight gain, which is beneficial in long-term recovery. In conclusion, more research, particularly, RCTs, is required to help shape an evidence-based refeeding guideline outlining target calorie intakes and rates of advancement to assist clinicians in the treatment of adolescents with AN and related eating disorders.
